# Hexamethylene amiloride induces lysosome-mediated cell death in multiple myeloma through transcription factor E3

**DOI:** 10.1038/s41420-024-02269-9

**Published:** 2024-12-18

**Authors:** Nianhui Yang, Zexuan Dong, Weihao Xiao, Suqi Deng, Yizhen Li, Lei Hua, Yue Li, Yingying Wu, Kexiu Huang, Wei Zhou, Hua Wang, Yonghua Li, Juan Du, Hui Zeng

**Affiliations:** 1 The First Affiliated Hospital of Jinan University, Guangzhou, Guangdong China; 2 Department of Oncology, Guangzhou Hospital of Integrated Traditional and Western Medicine, Guangzhou, Guangdong China; 3 Department of Hematology, Guangzhou First People’s Hospital, Institute of Blood Transfusion and Hematology, Guangzhou Medical University, Guangzhou, China; 4 Department of Hematologic Oncology, State Key Laboratory of Oncology in South China, Collaborative Innovation Center for Cancer Medicine, Sun Yat-sen University Cancer Center, Guangzhou, China; 5 Department of Hematology, General Hospital of Southern Theatre Command of PLA (People’s Liberation Army), Guangzhou, Guangdong China; 6 Department of Hematology, Guangdong Provincial People’s Hospital (Guangdong Academy of Medical Sciences), Southern Medical University, Guangzhou, China

**Keywords:** Myeloma, Myeloma

## Abstract

Multiple myeloma (MM) is the second common hematological malignancy characterized by the abnormal proliferation of plasma cells. Although advances in the past decades have led to improved outcomes and longer survival, MM remains largely incurable. New targets and targeted therapy may help to achieve better outcomes. Proton exporter NHE1 is highly expressed by tumor cells to maintain pH gradient for their survival and its inhibitor Hexamethylene amiloride (HA) has been demonstrated anti-tumor effect. However, whether HA could inhibit MM remains unknown. In this study, we firstly demonstrated that elevated expression level of NHE1 is associated with poor prognosis of MM. Moreover, the NHE1 inhibitor HA inhibited growth and induced apoptosis effectively in both MM cell lines and primary bone marrow cells from MM patients. Mechanistically, inhibitory effect was achieved partially through TFE3-mediated lysosomal production. With a MM xenograft mouse model, we verified that HA has a significant anti MM effect in vivo. Importantly, HA induced apoptosis of the carfilzomib-resistant MM cells and enhanced the effect of carfilzomib in MM. In summary, we demonstrated that NHE1 inhibitor HA can effectively inhibit MM growth both in vitro and in vivo, providing a new therapeutic strategy for improved outcome of de novo and resistant MM.

## Introduction

Multiple myeloma (MM) is a hematologic malignancy with the proliferation of abnormal clonal plasma cells in the bone marrow, leading to bone destruction, kidney insufficiency, anemia, and hypercalcemia. Approximately 588,000 people worldwide are diagnosed in MM every year [1–3]. In the last decades, significant progress has been made owing to the FDA approval of proteasome inhibitors including bortezomib, carfilzomib, and ixazomib, in addition to immunomodulatory drugs such as thalidomide, lenalidomide, and pomalidomide [4]. However, MM is still an incurable disease. Carfilzomib, the second-generation proteasome inhibitor, is an option for refractory MM patients [5], but there is still a number of patients develop resistance to carfilzomib treatment [6]. It is essential to reduce resistance or dosage of carfilzomib and find new targets to improve patients’ quality of life and clinical outcomes.

Na^+^/H^+^ exchanger 1 (NHE1) is one of the SLC9 transporter family members which function to regulate intracellular pH (pHi) by exchanging an intracellular proton for an extracellular sodium ion. It is associated with cell migration, cellular proliferation and control of cell volume [7, 8]. Elevated NHE1 emerges as a marker for tumorigenesis and prognosis and its increased activity results in intracellular alkalization and extracellular acidification that drives cancer progression [9–11]. Hexamethylene amiloride (HA) is a derivative of amiloride and a selective inhibitor of NHE1 [12]. HA has shown its anti-tumor effects, blocking NHE1 by HA decreases the pHi and induces apoptosis in leukemic cells [13]. HA treatment in acute myeloid leukemia cells also significantly increased their sensitivity to sorafenib treatment [14]. Interestingly, HA targets breast cancer cells by reactive oxygen species- and lysosome-dependent programmed necrotic mechanism [15]. However, the role of NHE1 and the therapeutic effects of its inhibitor in multiple myeloma remains unknown.

Herein, we investigate the effect of HA on MM and underlying mechanisms. We demonstrated that HA induced MM cell death. We also proved that HA has anti-MM effect in a xenograft mouse model and can overcome the carfilzomib resistance of MM cells and enhanced the effect of carfilzomib. This study may provide a new treatment strategy for clinical treatment of MM.

## Results

### Elevated NHE1 expression in multiple myeloma patients is associated with poor prognosis

**Fig. 1:**
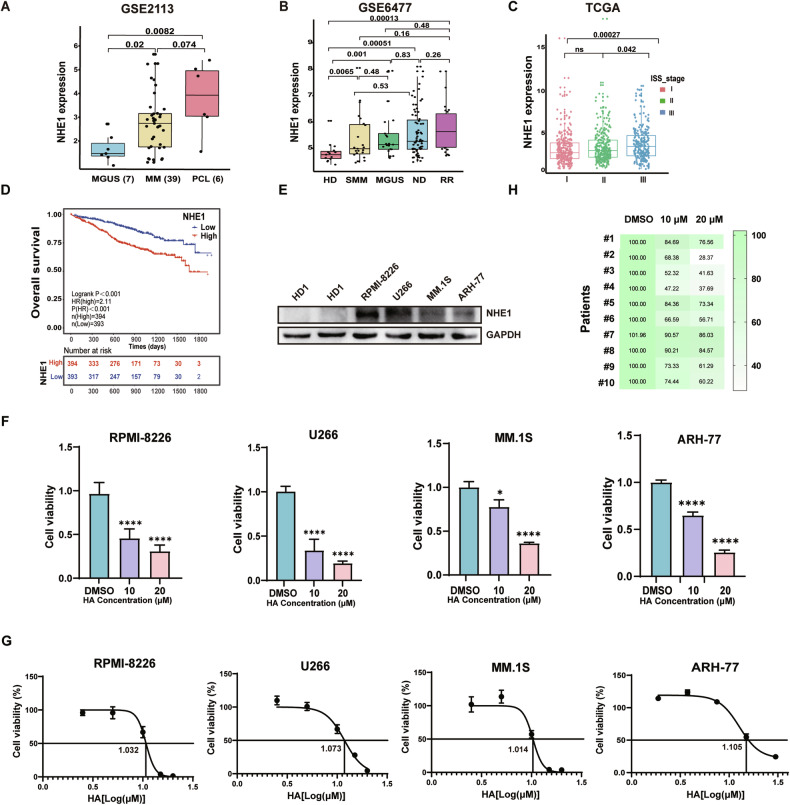
Elevated NHE1 expression in multiple myeloma patients is associated with poor prognosis. **A** Comparison of NHE1 expression levels between MGUS, MM and PCL in MM datasets (GSE2113). MGUS, monoclonal gammopathy of undetermined significance. MM, Multiple myeloma. PCL, plasma cell leukemia. **B** Comparison the expression levels of NHE1 in HD, SMM, MGUS, ND and RR in MM datasets (GSE6477). HD, healthy donor. SMM, smoldering multiple myeloma. ND, newly diagnostic multiple myeloma. RR, refractory and recurrence. **C** Analysis of NHE1 expression levels in different stages of MM in TCGA datasets. **D** The survival analysis of patients with high and low expression level of NHE1 in TCGA datasets. **E** Western blot of the expression of NHE1 of RPMI-8226, U266, MM.1S and ARH-77, normalized by GAPDH. HD (healthy donor). **F** The effect of NHE1 inhibitor HA on cell viability in RPMI-8226, U266, MM.1S and ARH-77 cell lines. Cells were treated with HA at indicated concentration for 48 h. **G** The IC50 of HA on cell viability in RPMI-8226, U266, MM.1S and ARH-77 cell lines. Cells were treated with HA at indicated concentration for 48 h. **H** The effect of HA on cell viability in ten primary MM patient cells. CD138+ cells were selected and treated with HA at indicated concentration for 48 h. Cell viability was assessed with CCK-8 kit. Data are presented as the mean ± SD, and differences were compared using the 2-tailed Student’s *t* test. *P < 0.05, ***P < 0.001, ****P < 0.0001, ns, not significant.

Elevated NHE1 emerges as a marker for tumorigenesis and prognosis [9, 10, 16]. We wonder whether the expression of NHE1 is elevated in MM patients. In order to investigate the expression level of NHE1 during progression of multiple myeloma, we analyzed the expression level of NHE1 in a gene expression omnibus (GEO) dataset (GSE2113). We found that the expression of NHE1 increased along with the progression of the disease from monoclonal gammopathy of undetermined significance (MGUS) to multiple myeloma (MM) stage (Fig. 1A). Moreover, in another GEO dataset (GSE6477), NHE1 is highly expressed in new diagnosis (ND) and refractory recurrence (RR) patients compare to health donor (HD) (Fig. 1B). We further analyzed the expression of NHE1 in the TCGA database, and found that NHE1 expression increased along with the aggravation of MM stage (Fig. 1C). Notably, elevated NHE1 expression was correlated with shorter survival of MM patients (Fig. 1D). Next, we compared the NHE1 expression level in four MM cell lines and in the bone marrow cells from a healthy donor, the result showed that NHE1 is overexpressed in MM cells (Fig. 1E). Next, we wonder whether targeting NHE1 could inhibit the growth of MM cells. To test the effect of NHE1 inhibition on MM cells, we treated the MM cell lines RPMI-8226, U266, MM.1S, and ARH-77 with HA, an inhibitor of NHE1. As shown in Fig. 1F, HA significantly decreased the viability of the MM cell lines, and the inhibition effect is dose-dependent. We also show the IC50 of HA in these four cell lines (Fig. 1G). To further explore the efficacy of HA on MM, we treated CD138+ cells obtained from the bone marrow of MM patients with HA and examined the viability of these primary MM cells, the patient information was shown in Table S4. Primary cells were treated with HA at 10 µM and 20 µM for 48 h and tested for viability. As shown in Fig. 1H, the cell viability was significantly reduced by HA in all tested primary bone marrow cells from ten patients. In addition, to further evaluate the effect of NHE1 inhibition on MM cells, we knock out NHE1 in RPMI-8226 cells. As expected, knockout of NHE1 decreased the viability and increase the percentage of apoptosis in RPMI-8226 cells (Fig. S1A–C). The knockout of NHE1 also decreased the intracellular pH and induced an increase of lysotracker signal as well as the permeability of the lysosome membrane in the RPMI-8226/NHE1 KO cells (Fig. S1D, E). Taken together, our data suggested that elevated level of NHE1 is associated with poor prognosis and targeting NHE1 with its inhibitor HA suppresses the growth of MM cell lines.

### HA induces apoptosis and decreased intracellular pH in MM cell lines

**Fig. 2:**
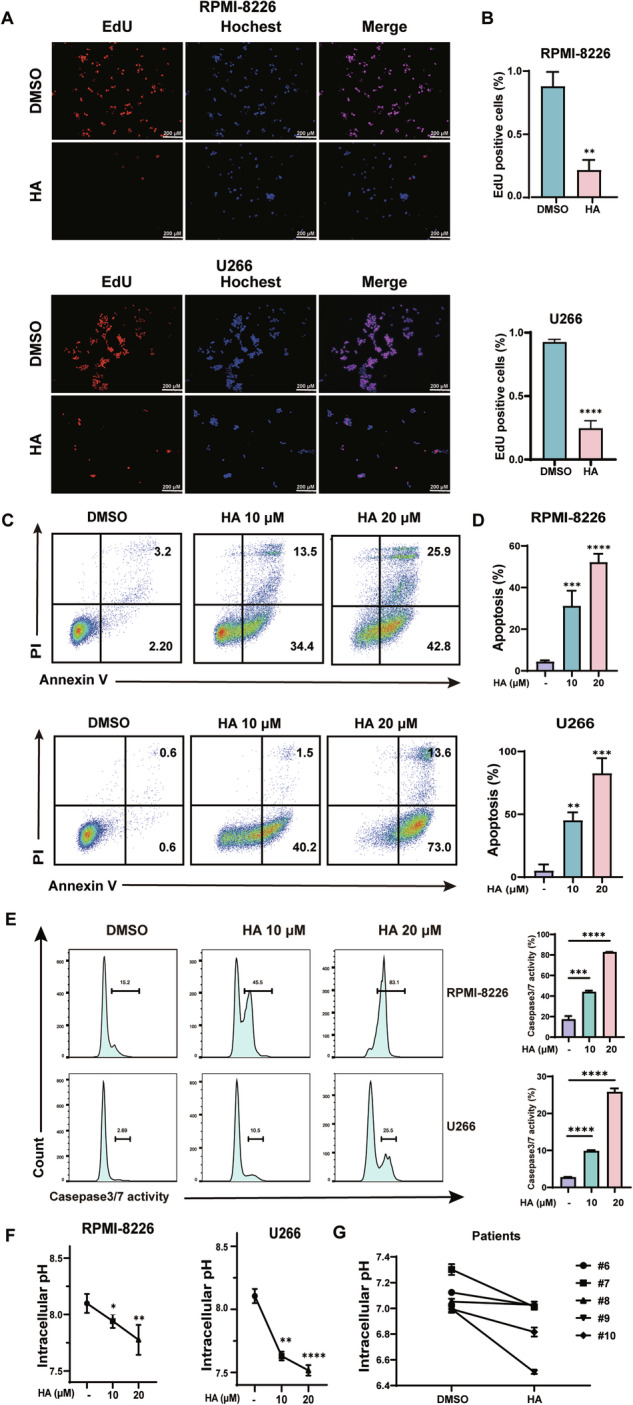
HA induces apoptosis and decreased intracellular pH in MM cell lines. **A** Edu detected cell proliferation after treated with HA for 48 h at 10 μM of RPMI-8226 (upper) and U266 (lower). **B** Statistical analysis of Edu positive cells of RPMI-8226 (upper) and U266 (lower). in (**A**). **C** Apoptosis assay of RPMI-8226 (upper) and U266 (lower) treated with HA at 10 μM and 20 μM for 48 h. The percentage of apoptotic cells (Annexin-V + PI + ) after treatment was analyzed by the flow cytometry. Representative scatter plots are shown. **D** Statistical analysis of apoptotic cells of RPMI-8226 (upper) and U266 (lower) in (**C**). Percentages of apoptotic cells of RPMI-8226 and U266 were calculated from three independent experiments. **E** Caspase3/7 activity positive cells of RPMI-8226 and U266 when treatment with HA for 48 h at 10 μM. **F** BCECF-AM probe detected the pHi of RPMI-8226 (left) and U266 (right) after treatment with HA at 10 μM for 48 h. **G** The effect of HA on pHi in primary MM patient cells treated with HA for 48 h.Data are presented as the mean ± SD of at least three independent experiments, and comparisons were evaluated by two-tailed Student’s *t* test. *P < 0.05, **P < 0.01, ***P < 0.001, ****P < 0.0001.

To investigate whether HA suppresses the proliferation of MM cells, RPMI-8226 and U266 were treated with HA at 10 µM and subjected to 5-ethynyl2′-deoxyuridine (EdU) incorporation assay. We observed that the proliferation of MM cells was significantly inhibited in the HA group compared to control (Fig. 2A, B). Moreover, to explore whether HA induces apoptosis of MM cell lines, RPMI-8226, U266 and MM.1S were exposed to HA (10 µM, 20 µM) for 48 hours and stained with PI and Annexin-V. As shown in Fig. 2C, HA induced apoptosis in 31.2% (average) of RMPI-8226 cells when treated with HA at 10 µM, and increased to 52.2% (average) at 20 µM. Similarly, HA induced cell apoptosis in 45% and 82.6% of U266 cells at 10 µM and 20 µM, respectively (Fig. 2D). Caspase-3 and Caspase-7 are critical to cell apoptosis, so we detected the percentage of Caspase-3/Caspase-7 by the Caspase-3/7 activity apoptosis assay kit. As shown in Fig. 2E, Caspase-3/7 positive cells were detected in 45.4% of RPMI-8226 cells and 10.5% in U266 at 10 µM, and the activity further increased to 83.1% of RPMI-8226 cells and 25.5% in U266 at 20 µM. Similar results were obtained for MM.1S cells (Fig. S2A, B). As NHE1 is a Na^+^/H^+^ exchanger, we examined whether HA treatment alters the intracellular pH (pHi). Interestingly, pHi decreased in the HA group for both RPMI-8226 and U266, indicating that the internal environment of the cells became more acidic (Fig. 2F). We next investigate whether HA also change pHi in primary MM cells as in MM cell lines. Interestingly, HA treatment also the decreased intracellular pHi in primary MM cells from all five patients tested (Fig. 2G). Collectively, these results suggest that HA inhibits proliferation, induces apoptosis, and induced intracellular acidification in MM cells.

### HA induces enhanced lysosome biogenesis and their rupture in MM

**Fig. 3:**
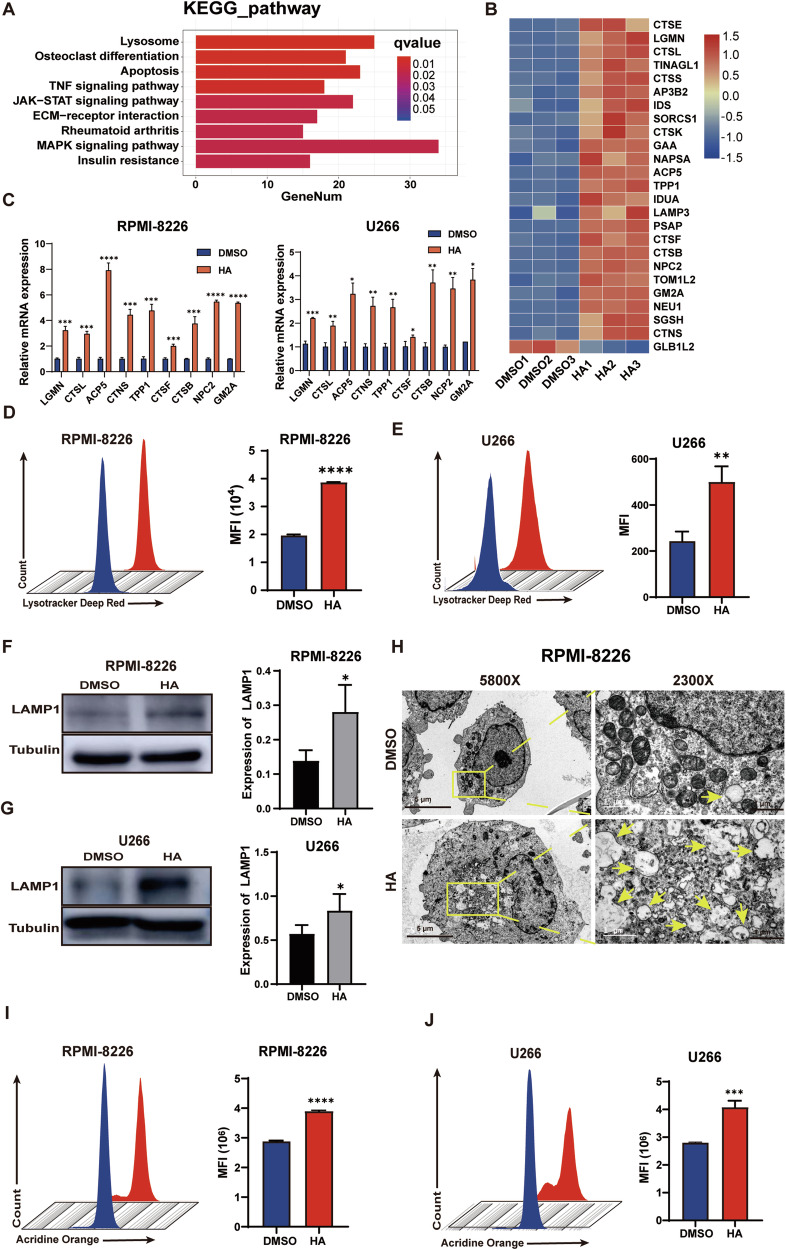
HA induces enhanced lysosome biogenesis and their rupture in MM. **A** KEGG analysis of the up-regulated genes. **B** Heatmap show Log2 fold change of the significantly differentially expressed genes in lysosomal pathway. **C** RT-qPCR of some significantly differentially expressed genes in lysosomal pathway of RPMI-8226 (left) and U266 (right). Cell were treated with HA at 10 μM for 48 h. **D**, **E** Lysotracker staining of RPMI-8226 (**D**) and U266 (**E**) after treated HA at 10 μM for 48 h. Right panel, mean fluorescence intensity statistics. **F**, **G** Western Blot detect the expression of LAMP1 of RPMI-8226 (**F**) and U266 (**G**) treated HA at 10 μM for 48 h and statistical analysis of LAMP1 expression, normalized by tubulin. **H** Transmission electron microscope shows the lysosome when RPMI-8226 treated HA for 48 h, yellow arrow: lysosome. **I**, **J** Acridine Orange staining of RPMI-8226 (**I**) and U266 (**J**) after treated HA at 10 μM for 48 h. Right panel, mean fluorescence intensity statistics. Data are presented as the mean ± SD of at least three independent experiments, and comparisons were evaluated by two-tailed Student’s *t* test. *, *P* < 0.05, **P < 0.01, ***P < 0.001, ****P < 0.0001.

To identify the underlying mechanism of the inhibitory effects of HA in MM cells, we performed transcriptomic profiling of U266 cells treated with HA for 48 h. 1651 differentially expressed genes (DEGs) were identified for the HA group compared to the control, including 1190 up-regulated genes and 461 down-regulated genes (Fig. S3A). Interestingly, lysosome was among the top 9 pathways in the KEGG analysis of the up-regulated genes (Fig. 3A). Most of the DEGs in the pathway were upregulated, including lysosomal hydrolase enzymes, CTSE coding Cathepsin E for example (Fig. 3B). To validate whether genes in the lysosome pathway were upregulated in HA-treated MM cells, we firstly determined the mRNA expression levels of the essential genes in HA-treated cells. Consistent with RNA-seq data, the expression of these selected genes involved in lysosome pathway were all significantly increased in both RPMI-8226 and U266 cells treated with HA (Fig. 3C). Since our data indicated that the lysosome pathway was activated, we hypothesized that the treatment of HA led to increased lysosome biogenesis. To detect whether the number of lysosomes was increased, we stained HA-treated MM cells with lysotracker dyes that labelled lysosomes with red fluorescence. The increased intensity indicated the quantity of lysosome increased significantly in HA-treated RPMI-8226, U266, MM.1S (Fig. 3D, E, Fig. S3B) and primary MM cells (Fig. S3C). To confirm whether the number of lysosomes increases in response to HA treatment, western blotting was performed to examine the expression level of LAMP1, a marker of lysosome, in HA-treated cells. The expression of LAMP1 was significantly increased in the HA group in both cell lines (Fig. 3F, G). To further investigate the changes of internal organelles in HA-treated cells, we fixed the cells and observed the alteration of organelles including lysosome with transmission electron microscopy (TEM). Consistently, we observed that the number of lysosomes significantly increased in the HA group. Interestingly, the lysosomes in the HA-treated MM cells were swelling (Fig. 3H). Therefore, we went on to test whether HA treatment might induce the rupture of lysosome. We measured the lysosomal membrane stability by AO staining, which emits green fluorescence when acid substances are released from damaged lysosomes. Interestingly, HA increased lysosomal membrane permeability, which could lead to the subsequent release of lysosomal enzymes and eventually induce cell apoptosis (Fig. 3I, J). Thus, these results showed HA induces excessive biogenesis of lysosome, but with increased permeability, which might lead to apoptosis of treated MM cells.

### HA-Induced MM cell death depends on TFE3

**Fig. 4:**
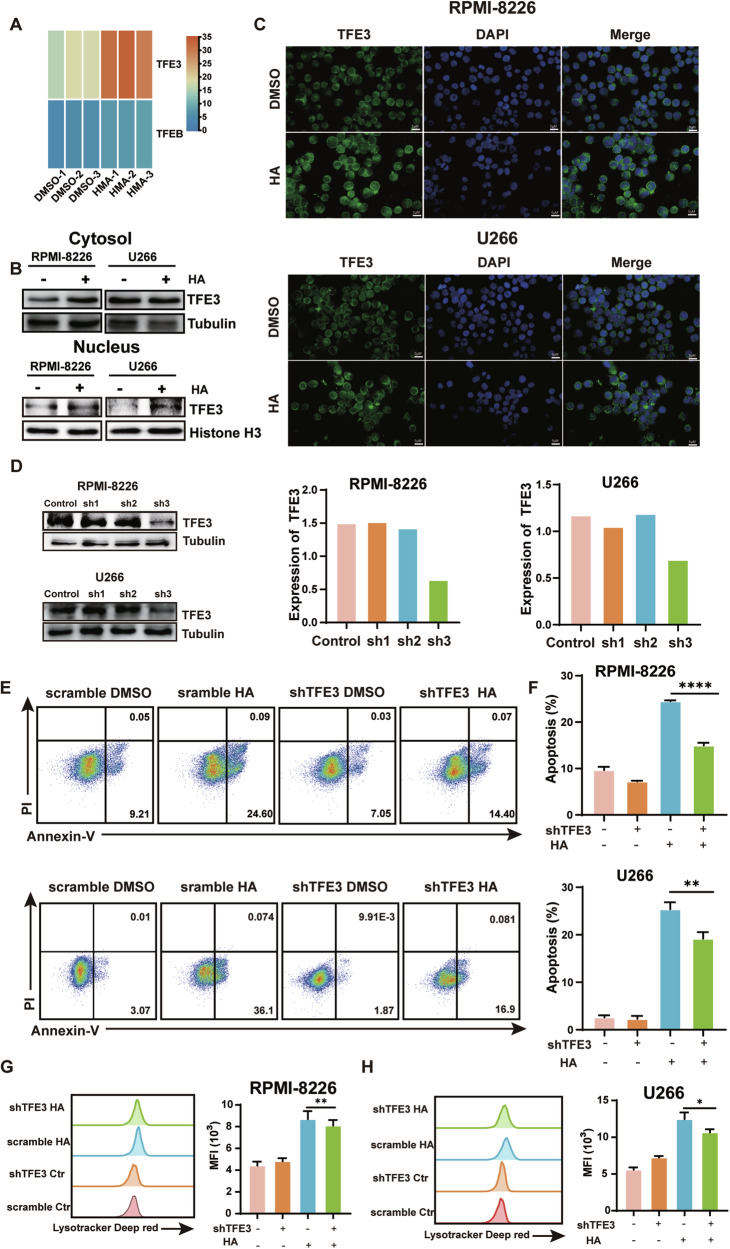
HA-Induced MM cell death depends on TFE3. **A** Analysing the TFE3 and TFEB expression in RNA-seq data. **B** Western Blot detecting the expression levels of TFE3 in cytosol and nucleus of RPMI-8226 and U266 treated HA at 10 μM for 48 h, normalized by tubulin or histone H3 respectively. **C** Immunofluorescence of the TFE3 in the nucleus in RPMI-8226 (upper) and U266 (lower) cells when treated with HA at 10 μM for 48 h. **D** Western Blot of the expression levels of TFE3 when infected with lentivirus carrying shTFE3 in RPMI-8226 and U266. **E** Apoptosis assay of RPMI-8226/TFE3 KD (upper) and U266/TFE3 KD (lower) treated with HA at 10 μM. **F** Statistical analysis of apoptotic cells for RPMI-8226/TFE3 KD cells (upper) and U266/TFE3 KD cells (lower) in (**E**). **G**, **H** Lysotracker assay detecting the lysosome of RPMI-8226/TFE3 KD (**G**) and U266/TFE3 KD (**H**) treated with HA at 10 μM for 48 h. Data are presented as the mean ± SD of at least three independent experiments, and comparisons were evaluated by two-tailed Student’s *t* test. *P < 0.05, **P < 0.001, ****P < 0.0001.

It has been well acknowledged that TFE3 and TFEB are master regulators for lysosomal biogenesis by driving the expression of lysosomal-related genes [17, 18]. Therefore, we firstly examined whether the expression of TFE3 or TFEB increased in our RNA-seq analysis (Fig. 4A). We found that the basic expression value of TFE3 is higher than TFEB and the expression level increased with HA treatment and we verified that by RT-qPCR (Fig. S4A, B). Next, we determined whether HA treatment leads to nuclear translocation of TFE3. We first used western blot to detect the TFE3 expression in the cytosol and nucleus. The expression levels of TFE3 in both compartments were increased with HA treatment compared with control (Fig. 4B). Moreover, immunofluorescence staining showed significant enhancement of nuclear translocation of TFE3 in HA-treated RPMI-8226 and U266 cells (Fig. 4C). TFE3 is phosphorylated by mTOR and locates in the cytoplasm [19, 20]. When the cell is stimulated, TFE3 is dephosphorylated and activated, and subsequently translocated to the nucleus to promote autophagy and lysosomal gene transcription, leading to lysosome biogenesis. Therefore, we examined the mTOR activity by western blotting, the phosphorylated mTOR was decreased when treated with HA (Fig. S4C). Furthermore, to further validate whether TFE3-mediated lysosome activation contributes to HA-induced cell death, we generate MM cells stably knockdown TFE3 expression. We first verified the knockdown of TFE3 expression at the protein level. The results showed that TFE3 shRNA infection effectively down-regulated TFE3 at protein level. RPMI-8226/shTFE3#3 and U266/shTFE3#3 were selected for further examination (Fig. 4D). More importantly, TFE3 knockdown could partially restore cell viability in the presence of HA (Fig. S4D, E). In addition, TFE3 knockdown decreased HA-induced apoptosis (Fig. 4E, F). Intriguingly, we also found that TFE3 knockdown significantly decreased the intensity of lysotracker, indicating attenuated lysosome biogenesis compared to the HA group (Fig. 4G, H). Taken together, these results confirmed that HA induces cell death partially through TFE3-mediated lysosome biogenesis.

### Antimyeloma efficacy of HA in vivo

**Fig. 5:**
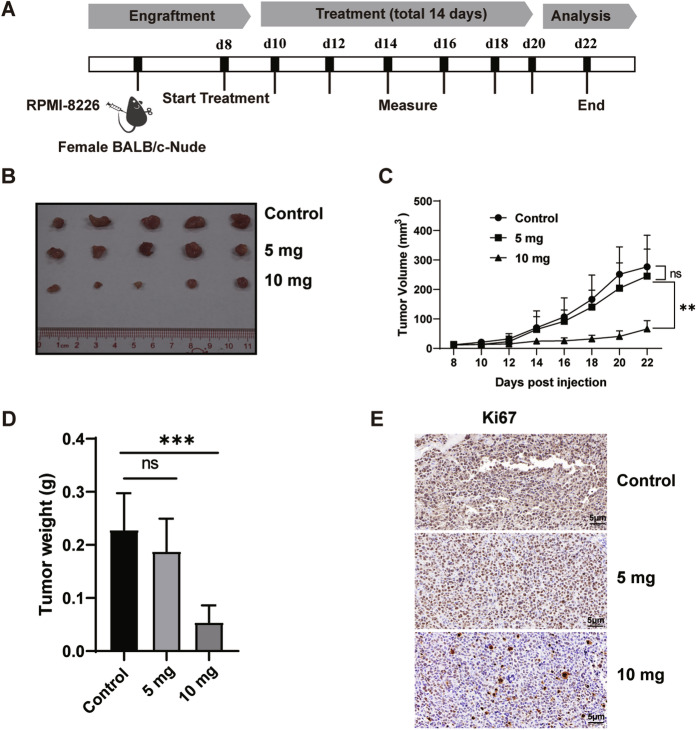
Antimyeloma efficacy of HA in vivo. **A** Schematic diagram of the experimental design. Mice with palpable tumors were randomly divided into three groups. Vehicle treatment group was used as the control (*n* = 5), and the other two were intraperitoneal injection HA at the dosage of 5 or 10 mg/kg body weight (*n* = 5 each group) once daily for consecutive ten days. **B** Images of tumors of HA treatment groups and the control group. **C** The tumor volume curves of the HA treatment groups and the control group. Tumor volumes were measured every other day. Tumor volume = (tumor length × width^2^)/2. The day 22 post injection RPMI-8226 tumor volume test was performed to determine statistical significance. **D** The tumor weights at day 22 post injection RPMI-8226 test was performed to determine statistical significance. **E** The expression of Ki67 between control and HA. One representative image is shown per group. **P < 0.01, ***P < 0.001, ns, not significant.

We have proven that HA induces MM cell apoptosis effectively in vitro. We then wondered whether HA had an anti-myeloma effect in vivo. We established subcutaneous MM mouse model by injecting s.c. RPMI-8226 cells into BALB/c Nude mice. When tumors were palpable, mice were randomly divided into three groups (HA 5 mg group, HA 10 mg group and control group, each group *n* = 5) followed by administration of either vehicle or HA (5 mg/kg/day and 10 mg/kg/day). The MM mouse model was testified by immunohistochemistry staining of human CD138 (Fig. S5A). Tumor volumes and mice body weights were examined every other day (Fig. 5A). The results showed that the tumor volume of the HA 10 mg group was significantly decreased, but a minimal change of tumor volume was observed in the 5 mg group (Fig. 5B, C), indicating efficient inhibition of MM tumor with HA at 10 mg/kg/day dose. Consistently, the weights of tumor were also significantly decreased in 10 mg group (Fig. 5D). Interestingly, HA treatment didn’t show a prominent effect on mice body weights, alanine aminotransferase, total protein and creatine kinase (Fig. S5B, C). To further confirm the inhibition effect, immunohistochemistry was performed to detect the proliferation of MM tumor cells. As shown in Fig. 5E, HA inhibited the proliferation of tumors remarkably in the 10 mg group. Therefore, all results collectively demonstrated that the HA exerted potent anti-MM activity in vivo.

### HA overcomes carfilzomib resistance and enhances the antimyeloma effects of carfilzomib

**Fig. 6:**
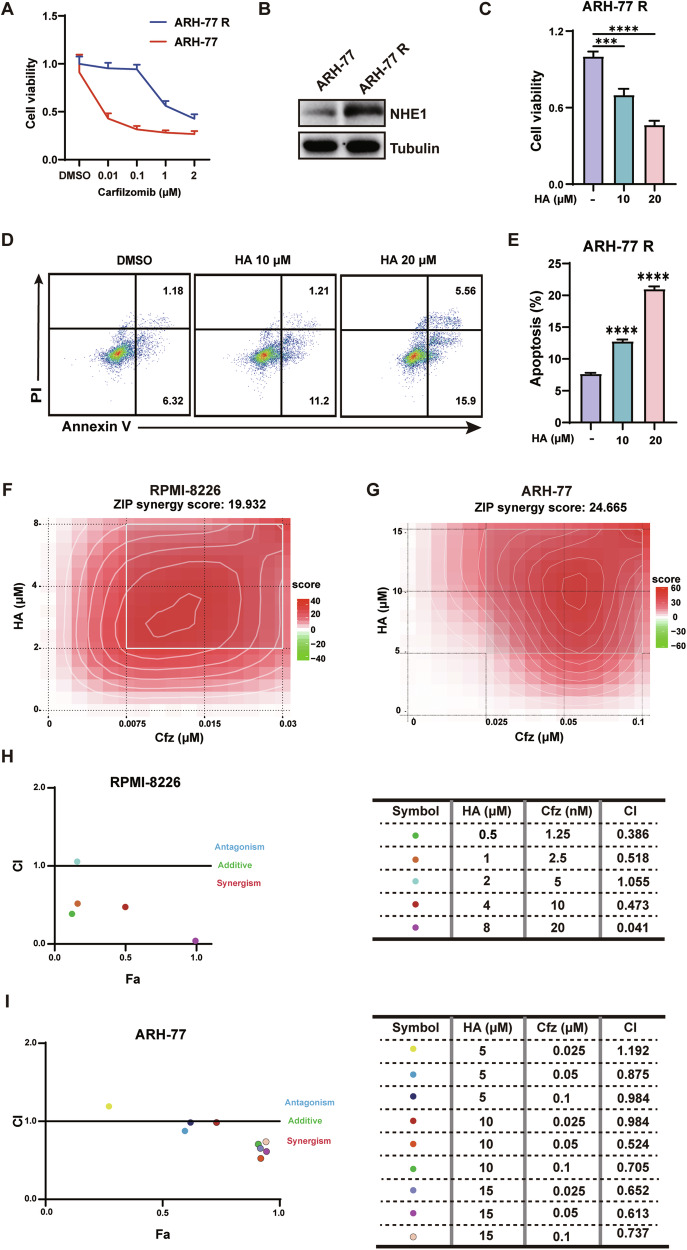
HA overcomes carfilzomib resistance and enhances the antimyeloma effects of carfilzomib. **A** The cell viability of carfilzomib-resistant cell line ARH-77 R and parental cell line ARH-77 treated with carfilzomib for 48 h at the indicated concentration of carfilzomib. **B** Western Blot of the expression levels of NHE1 in ARH-77 R and parent cell line ARH-77, normalized by tubulin. **C** The cell viability of ARH-77 R treated with HA at the indicated concentration for 48 h. **D** Apoptosis assay of ARH-77 R treated with HA at 48 h. The percentage of apoptotic cells (Annexin-V + PI + ) after treatment was analyzed by the flow cytometry. **E** Statistical analysis of apoptotic cells of ARH-77 R in (**D**). Percentages of apoptotic cells of ARH-77 R were calculated from three independent experiments. **F** Cell viability of RPMI-8226 cells. Cells were treated with increasing concentration of carfilzomib (Cfz, 0.075 µM, 0.015 µM, 0.03 µM) and HA (2 µM, 4 µM, 8 µM) alone or in combination for 48 h. **G** Cell viability of ARH-77 cells were treated with carfilzomib (Cfz, 0.025 µM, 0.05 µM, 0.1 µM) and HA (5 µM, 10 µM, 15 µM) alone or in combination for 48 h. Cell viability was performed by CCK-8 assay. Synergy was assessed by the Synergy Finder website (https://synergyfinder.fimm.fi/). **H**, **I** Combination Index (CI) of HA and carfilzomib in RPMI-8226 or ARH-77 cells. The CI of HA and carfilzomib was calculated by Compusyn software. CI < 1, CI = 1, and CI > 1 indicate synergistic, additive, and antagonistic effects respectively. Data are presented as the mean ± SD of at least three independent experiments, and comparisons were evaluated by two-tailed Student’s *t* test. ***P < 0.001, ****P < 0.0001.

Next, to investigate whether HA could overcome drug resistance in MM, we chose a carfilzomib-resistant cell line ARH-77 R. This cell line demonstrated resistance to carfilzomib at concentrations lower than 0.1 µM (Fig. 6A). Then we examined the expression of NHE1 in ARH-77 R, and found that NHE1 was highly expressed in ARH-77 R compared with ARH-77 (Fig. 6B). Therefore, we treated ARH-77 R with HA, wondering whether HA could reverse resistance to carfilzomib of this cell line. Interestingly, HA significantly inhibited the viability of ARH-77 R in a dose-dependent manner (Fig. 6C). Consistently, HA induced apoptosis significantly in ARH-77 R, demonstrating that HA has a fine prospect of overcoming drug resistance (Fig. 6D, E). Moreover, we wonder whether HA could enhance the therapeutic effect of carfilzomib as a combinatory candidate. Different concentrations of HA and carfilzomib alone or in combination was added in RPMI-8226 and ARH-77, and the viability of these treated cells were tested. As shown in Fig. 6F, G, synergistic effects were observed in both cell lines. To further validate the synergistic effects of HA and carfilzomib, we calculated the combination index (CI) using CompuSyn software after treating RPMI-8226 and ARH-77 cell lines with HA and carfilzomib alone or in combination. The CI value ranged from 0.041 to 1.055 in RPMI-8226 cells and 0.524 to 1.192 in ARH-77 cells, indicating a synergistic anti-proliferative effect for treatment with HA and carfilzomib in most combinations we tested, although in some concentrations the combination presented nearly additive and slightly antagonized effects (Fig. 6H-I). Collectively, these data suggest that HA overcame carfilzomib resistance and enhance its therapeutic effects.

## Discussion

As the most well-characterized isoform of the NHE family, NHE1 maintains the balance of intracellular and extracellular H^+^ [21]. Tumor cells enhance the utilization of glucose, glutamine, and other substances to produce a large amount of lactic acid and H^+^, and MM is no exception [22]. Tumor cells have a powerful mechanism to transport H^+^ out of the cell, leaving the cell in an alkaline state that is conducive to survival [23]. In our study, we find NHE1 is overexpressed in MM patients compare to healthy donors, and NHE1 expression increases with the severity of MM. These data suggest the potential important role of NHE1 in MM. In consistence with this, NHE1 inhibitor HA could significantly inhibit the proliferation of MM cells and induce apoptosis. In other cancers, HA can induce tumor cell apoptosis and activate autophagy in a caspase-dependent manner [24]. In MM, HA can also activate Caspase3/7 and reduce the pHi of MM cells. This is consistent with the role of HA to alter pHi and inhibit cell growth in AML [11].

In further exploration of the mechanism, we found that HA induced an increase of lysosomes, altered their membrane permeability, and increased TFE3 transport into the nucleus to activate lysosomal biogenesis. Lysosomes contain more than 60 kinds of hydrolases, including protease, peptidase, phosphatase, nuclease, glycosidase, sulfatase and lipase. Among them, the cathepsin family is of importance. When the permeability of the lysosomal membrane changes, these enzymes will be released into the cytoplasm, causing lysosomal membrane permeability (LMP) and leading to cell death. In other cases, LMP and cathepsin release are involved in the activation of effectors such as ROS, Bax, and iron, leading to other types of cell death, such as apoptosis, pyroptosis, and ferroptosis [25–27]. In this study, we observed increased LMP in the HA-treated MM cells. The intracellular acidification and enhanced lysosome biogenesis were also observed in primary MM cells. These collectively support lysosome biogenesis is involved in the HA-induced cell death of MM cells.

In addition, genes responsible for lysosomal generation were activated. TFE3 was shown to be the key player for HA-induced lysosome biogenesis in MM cells. After knocking down TFE3, the effect of HA on MM was partially salvaged, but could not be fully restored. Therefore, there could be other mechanisms contributing to HA-induced cell death in MM. Moreover, other cell death pathways involving lysosome in addition to apoptosis may also participate in HA-induced cell death in MM cells.

We have also noticed that HA could act as a weak P2X7 antagonist in RPMI-8226 cells and impair ATP-induced dye uptake [28], which may impact its application in treating MM. A preliminary assessment of the effects of HA in the presence of ATP indicates that HA doesn’t seem to counteract ATP as an antagonist of P2X7 (Fig. S6A–C). Moreover, the addition of ATP may compromise the inhibitory effect of HA. In addition, we genetically knocked out NHE1 in RPMI-8226 cells and observed similar results as HA treatment. These results collectively suggest that HA behaved as a NHE1 inhibitor rather than a P2X7 antagonist in our study. However, considering the complexity of the microenvironment of MM cells in clinical settings, the therapeutic effect of HA both as a NHE1 inhibitor and a P2X7 antagonist needs to be comprehensively evaluated.

In this study, we demonstrated that HA had an anti-tumor effect in vivo. The tumor volume in the experimental group was significantly smaller than that in the control group, and the expression of the proliferation marker Ki67 was also significantly reduced compared to the control group. Further exploration demonstrated that HA had the same inhibitory effect on the primary cells of MM patients. It has been reported that amiloride has a synergistic effect with dexamethasone, melphalan, etc. [29], and HA’s inhibition on MM is significantly stronger than amiloride. As we expected, HA in combination with carfilzomib achieved a better therapeutic effect than either alone. Notably, HA demonstrated an inhibitory effect on carfilzomib-resistant cells. These results suggest that HA could be considered as a candidate for the treatment of carfilzomib resistant patients or as a combinatory regime to achieve better therapeutic effects in MM. In addition, there is potential for the effectiveness of HA in combination with other anti-myeloma drugs, including lenalidomide, anti-CD38 antibodies, and steroids. Further studies exploring the combination of HA with anther anti-myeloma drug are warranted. Moreover, although studies are reporting the toxicity profiles and pharmacokinetics of HA with pre-clinical models [30–32], the safety and efficacy of HA need to be further explored in humans with phase I and II clinical trials, especially for the treatment of hematological diseases including MM.

In summary, this study found that HA inhibits MM in vitro and in vivo. HA is also effective in primary MM cells and could collaborate with carfilzomib. This study revealed the inhibitory effect of NHE1 inhibitor HA on MM and its potential mechanism, providing a new therapeutic choice for clinical treatment of MM.

## Material and method

### Cell lines and reagents

The MM cell lines RPMI 8226, MM.1S, ARH-77, ARH-77 R and U266 were cultured in RPMI 1640 medium (BI). ARH-77 R was kindly provided by Dr. Bingzong Li (The Second Affiliated Hospital of Soochow University, Suzhou, China). Human embryonic kidney cells (HEK293T) were cultured in Dulbecco’s Modified Eagle’s Medium (DMEM) with high glucose. All the media were supplemented with 10% fetal bovine serum (BI) and 100 U/mL penicillin, and 100 μg/mL streptomycin (Gibco). All cell lines were tested negative for mycoplasma with a Mycoplasma PCR detection kit (GeneCopoeia, USA) according to the manufacturer’s instructions. Cells were grown in a humidified atmosphere containing 5% CO2 at 37 °C, Hexamethylene amiloride (HA) was purchased from MedChemExpress (Shanghai, China).

### Human samples

Human bone marrow (BM) samples from MM patients were collected according to a protocol approved by the Institutional Review Board of The First Affiliated Hospital of Jinan University, with patients informed consent acquired in accordance with the Declaration of Helsinki. Mononuclear cells were isolated by density-gradient centrifugation (Ficoll-Paque, Solarbio, China) and followed by magnetic separation using CD138+ antibody-specific microbeads according to the manufacturer’s protocol (Miltenyi Biotech). Finally, the CD138+ cell was treated with DMSO and HA in Iscove’s Modified Dulbecco Medium (IMDM) supplemented with 20% fetal bovine serum (BI) and 100 U/mL penicillin, and 100 μg/mL streptomycin (Gibco) for 48 h. Cell viability was assessed using the cell counting kit-8 (CCK-8) (Biosharp, Guangdong, China) according to the manufacturer’s instructions.

### Quantitative real-time PCR (qRT-PCR)

Total RNA was isolated using Trizol reagent (Invitrogen, CA, USA). cDNA was then synthesized from 1 μg of total RNA with oligo(dT) primers using the HiScript II Q RT SuperMix (Vazyme, Nanjing, China). Quantitative real-time PCR (qRT-PCR) was implemented using the ChamQ SYBR qPCR Master Mix (Vazyme, Nanjing, China). The relative quantity of each transcript was determined using the relative standard curve method. The values were normalized to invariant control *GAPDH* expression. All primers for each gene are listed in Supplemental Table 1.

### Lentiviral production and infection

HEK 293 T packaging cells were seeded into 10 cm plates and while grow to 80% density replace with the culture medium without serum and antibiotics, after two hours, 8ug of scramble shRNA or TFE3 shRNAs were transfected into HEK 293 T cells, together with 6ug of pCMV-Gag-Pol and 4ug of pCMV-VSVG for lentivirus packaging using Lipo2000 reagent (Biosharp, Guangdong, China). After 12 h transfection, cells were re-fed with fresh medium. After 48 h and 72 h, medium containing virus was harvested and passed through 0.45 μm filters. MM cells were infected using ultra-centrifugation at 1000 rpm at 4 °C for 1 h with crude viral supernatant in the presence of 8 μg/ml polybrene (Biosharp, Guangdong, China), and then incubated in 5% CO_2_ at 37 °C for 12–16 h. After culture in fresh medium for 48 h, infected cells were selected with puromycin dihydrochloride (2 μg/ml) for at least 5 days, followed by subsequent assays. All plasmid vectors used in this study are listed in Supplemental Table 2.

### Cell viability assay

MM cell were seeded at a density of 5 × 10^4^ /ml in 96-well plates. Cells were treated with HA at indicated concentration (MedChemExpress, Shanghai, China). Forty-eight hours later, cell proliferation rates were subsequently assessed using the cell counting kit-8 (CCK-8) (Biosharp, Guangdong, China) according to the manufacturer’s instructions. HA were dissolved at 40 mM in dimethyl sulfoxide (DMSO). Cells in the control group were treated with vehicle (DMSO in RPMI-1640 supplemented with 10% FBS). Cell proliferation was observed using a Cell Light 5-ethynyl-2′-deoxyuridine (EdU) imaging kit (Beyotime, Shanghai, China) according to the manufacturer’s instructions, and cells were observed under a fluorescence microscope.

### Annexin-V / Propidium iodide staining

Cells were washed twice with cold PBS and then resuspended with 1× Binding Buffer, followed by adding with 5 μl Annexin-V and 10 μl propidium iodide (Yeasen Biotechnology) in 100 μl cell mixture (1 × 10^5 cells). The mixture was gently vortexed and incubated for 15 min at RT (25 °C) in the dark. After adding with 400 μl of 1× Binding Buffer, the cell solution was analyzed by flow cytometry. The percentage of apoptotic cells were calculated basing on cells labelled with both Annexin-V and PI.

### Caspase 3/7 activity assay

RPMI-8226 or U266 cells were cultured in 96-well plates for 48 h after exposure to the HA, caspase 3/7 activity was measured by a Cell Meter^TM^ caspase 3/7 active apoptosis assay kit (AAT Bioquest, CA, USA) following the manufacturer’s instructions using a 488 nm laser with 530/30 (GREEN) by FACSCanto II (BD Biosciences).

### RNA-seq analysis

U266 cells were treated in triplicate with DMSO or 10 μM HA for 48 h. A total of 6 samples (three for each group) were sequenced using the Illumina NovaSeq 6000 platform. Differentially expressed genes (DEGs) were identified using the DESeq2 package. Genes demonstrating altered expression with a false-discovery rate of *P* < 0.01 and greater than 2-fold change were considered differentially expressed.

### Myeloma models in nude mice

The human MM cell line RPMI-8226 were s.c. injected at a density of 1 × 10^7^ cells in pbs per site into female BALB/c Nude mice (4–6 weeks old, GemPharmatech, Guangdong, China). When tumors were measurable, mice were randomly divided into three groups. One group took vehicle as the control, and mice in the other two were intraperitoneally injected with HA at a dosage of 5 or 10 mg/kg body weight once daily until reaching the end of the experiment. Body weight and tumor volumes were monitored every other day (tumor volume = (tumor length × width^2^)/2). At the end of the experiment, the tumor volume of the experimental group was compared with that of the control group. Mice were euthanized and tumor samples were collected for further studies.

### Western blot analysis (WB)

Whole cell lysates were prepared from cells of interest as, Cell lysates were then clarified by centrifugation at 12,000 × g for 10 min and the protein concentrations in the supernatant were determined with a BCA protein assay kit (Beyotime). Equal amount of proteins was subjected to be fractionated by SDS polyacrylamidegel electrophoresis followed by transfer to polyvinyl idenedifluoride membranes. The blots were incubated with specific antibodies, followed by incubation with secondary horseradish peroxidase (HRP)-conjugated goat anti-mouse or anti-rabbit IgG (Beyotime) and detection was performed by using the Enhanced Chemical Luminescence reagent and methodology (Beyotime).

### Statistical analysis

All statistical analyses were performed on GraphPadPrism 8 (GraphPadSoftware, La Jolla, CA, USA). Data are expressed as mean ± standard error. The comparison between the two groups was conducted using the Student’s *t* test. *P* values less than 0.05 were considered statistically significant.

## Supplementary information

Supplemental Material

Supplemental Material

## Data Availability

The data supporting this study’s findings are available from the corresponding author upon request.
