# Supplementary material for: Hexamethylene amiloride induces lysosome-mediated cell death in multiple myeloma through transcription factor E3
Source: Cell Death Discov. 2024 Dec 18;10:505. doi: 10.1038/s41420-024-02269-9 (PMC11655536; doi:10.1038/s41420-024-02269-9)
Supplement: Supplementary file 1 — Supplemental Material [file 41420_2024_2269_MOESM1_ESM.docx]

**Supplementary figures and tables**

**
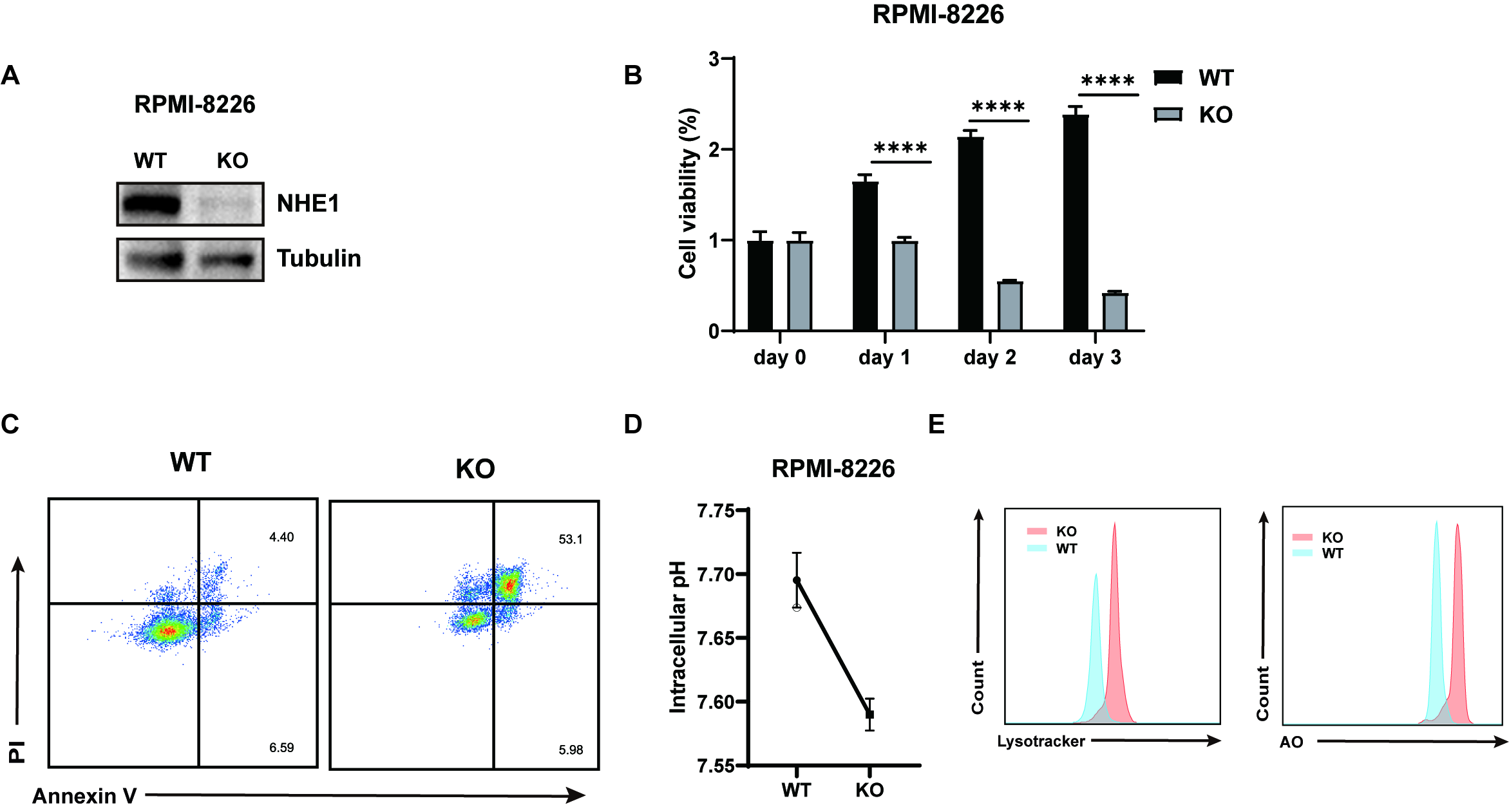
**

**Supplementary Fig. 1** Effect of NHE1 knockdown in RPMI-8226 cells. (A) The knockout efficiency of NHE1 (NHE1 KO) by western blotting in RPMI-8226. (B)Effects of NHE1 KO on the proliferation of RPMI-8226. (C)Apoptosis assay of RPMI-8226/NHE1 KO or RPMI-8226. (D) The effect of NHE1 KO on pHi in RPMI-8226/NHE1 KO or RPMI-8226. (E) Lysotracker (left) and AO (right) staining of RPMI-8226/NHE1 KO or RPMI-8226. Data are presented as the mean ± SD of at least three independent experiments, and comparisons were evaluated by two-tailed Student’s t test. ****, *P* < 0.0001.


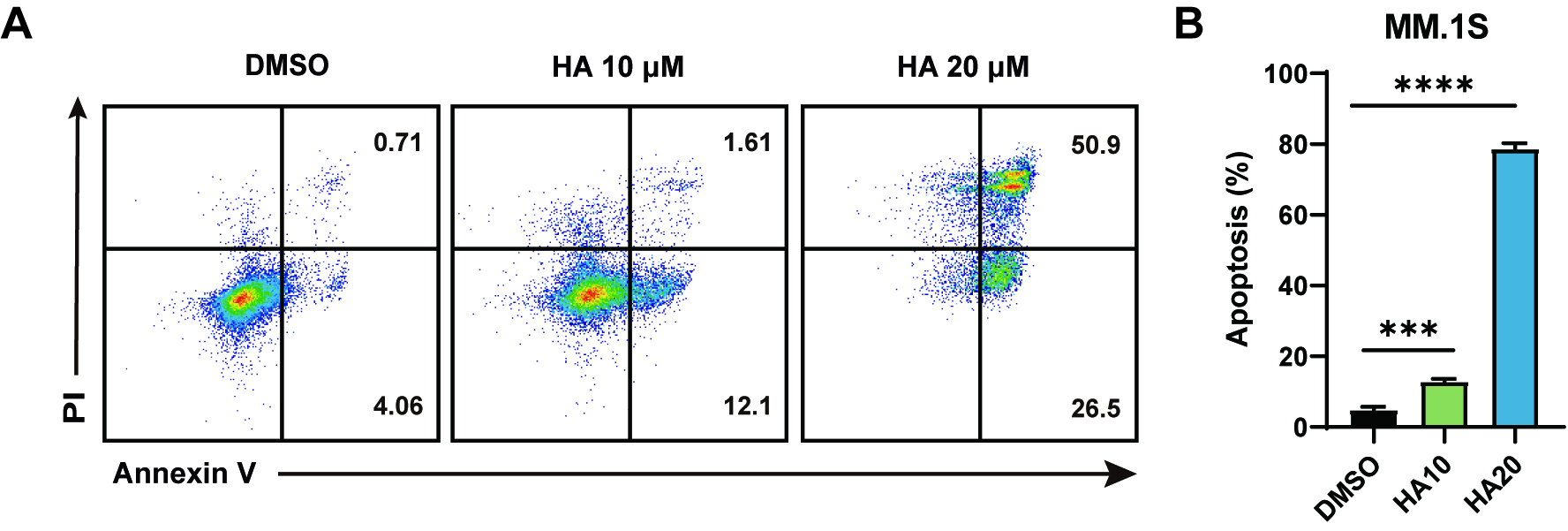


**Supplementary Fig. 2** HA induces apoptosis and decreased intracellular pH in MM cell lines. (A-B) Apoptosis assay of MM.1S treated with HA at 48h. The percentage of apoptotic cells (Annexin-V^+^PI^+^) after treatment was analyzed by the flow cytometry. Representative scatter plots are shown, and statistical analysis of apoptotic cells of MM.1S, percentages of apoptotic cells of MM.1S were calculated from three independent experiments. ***, *P* < 0.001, ****, *P* < 0.0001.


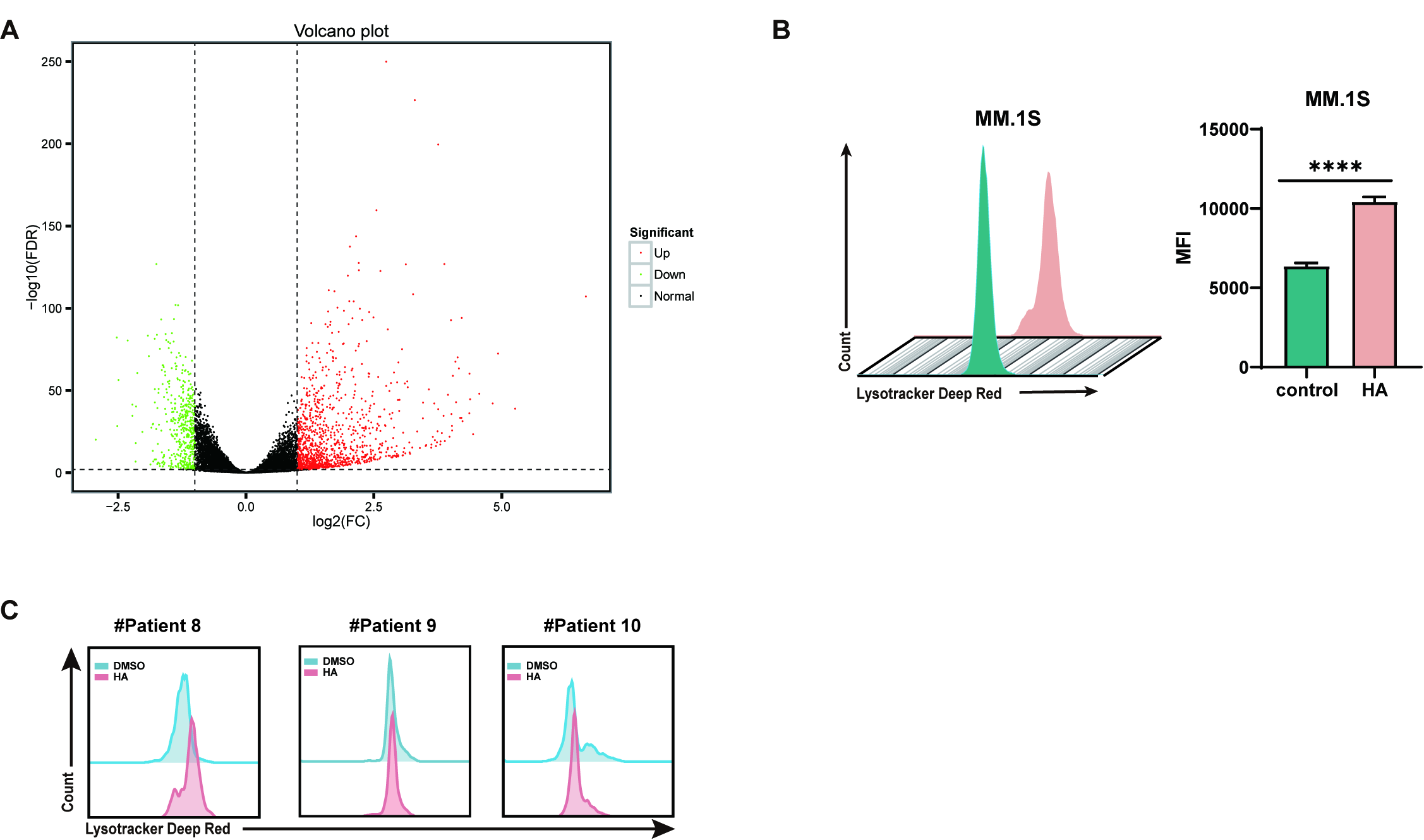


**Supplementary Fig. 3** HA-Induced MM cell death depends on TFE3. (A) Differentially expressed genes volcano map from RNA-seq in U266 cells. (B) Lysotracker staining of MM.1S after treated HA at 10 μM for 48h. Right panel, mean fluorescence intensity statistics. (E) Lysotracker staining of primary MM cells after treated HA at 10 μM for 48h. Data are presented as the mean ± SD of at least three independent experiments, and comparisons were evaluated by two-tailed Student’s t test. ****, *P* < 0.0001, ns, not significant.


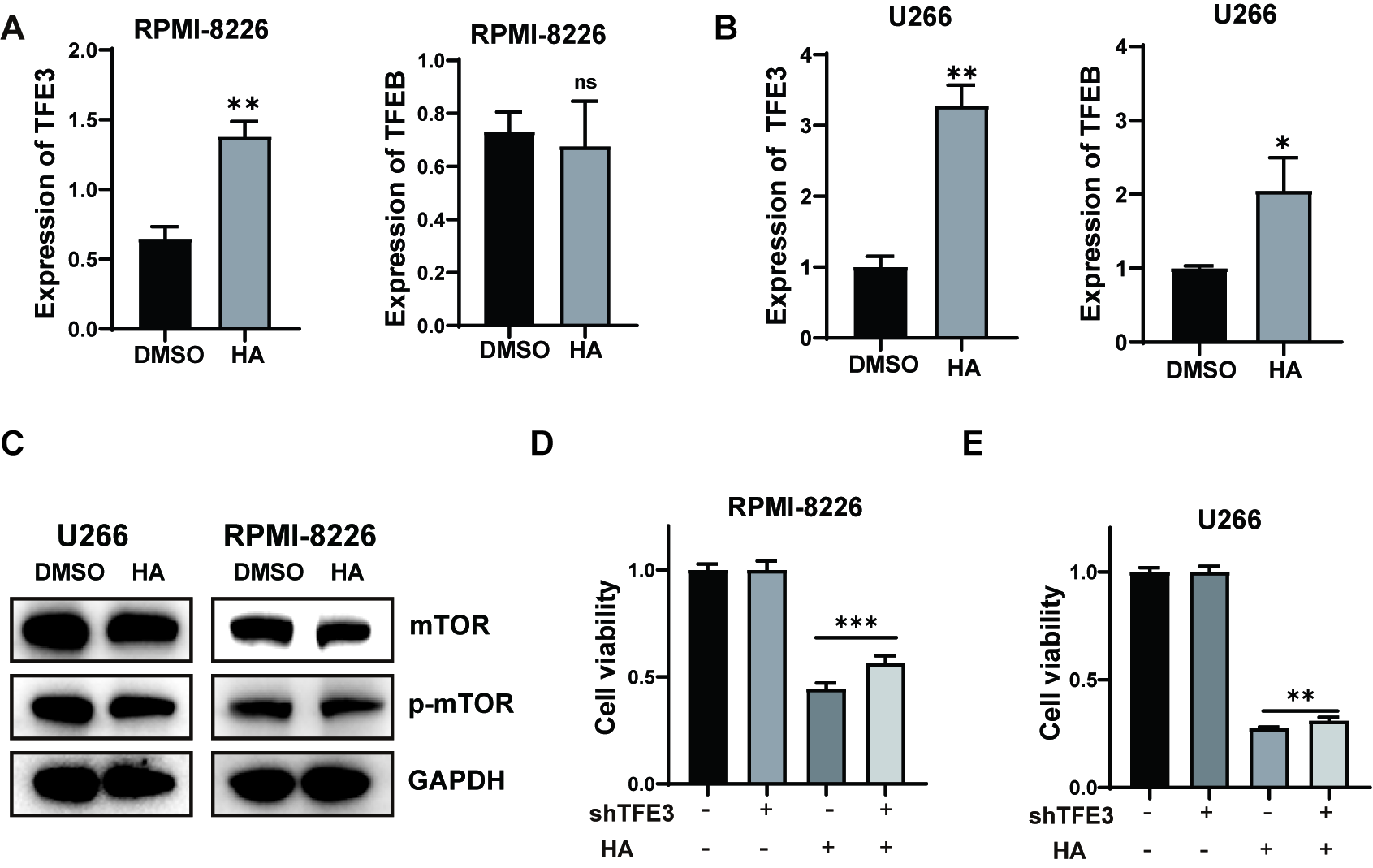


**Supplementary Fig. 4** HA-Induced MM cell death depends on TFE3. (A-B) RT-qPCR for TFE3 and TFEB when treated with HA in RPMI-8226 (A) and U266(B). (C) Western Blot detect the expression of mTOR and phosphorylated mTOR (p-mTOR) of RPMI-8226 and U266 treated HA at 10 μM for 48h, normalized by GAPDH. (D) Detecting the cell viability treated with HA when RPMI-8226 knockdown TFE3. (E) Detecting the cell viability treated with HA when U266 knockdown TFE3. *, *P* <0.05, **, *P* < 0.001, ***, *P* < 0.001, ns, not significant.


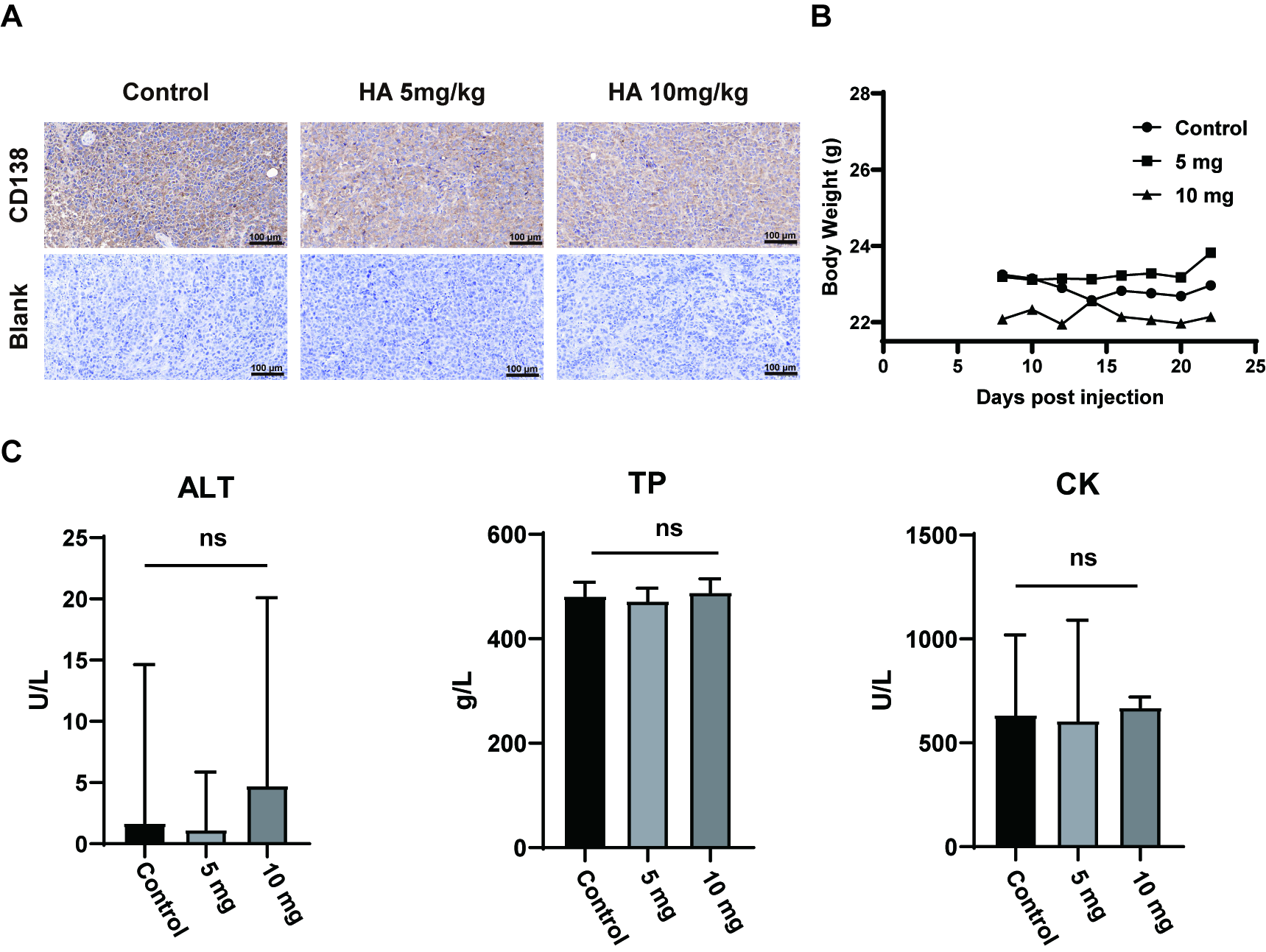


**Supplementary Fig. 5** Antimyeloma efficacy of HA *In vivo*. (A) Immunohistochemistry staining of human CD138 to testify the MM mouse model, Scale bar, 100 µm. (B) Average weight of mice after treatment with HA.

1. Alanine aminotransferase (ALT), total protein (TP) and creatine kinase (CK) of mice after treatment with HA. ns, not significant


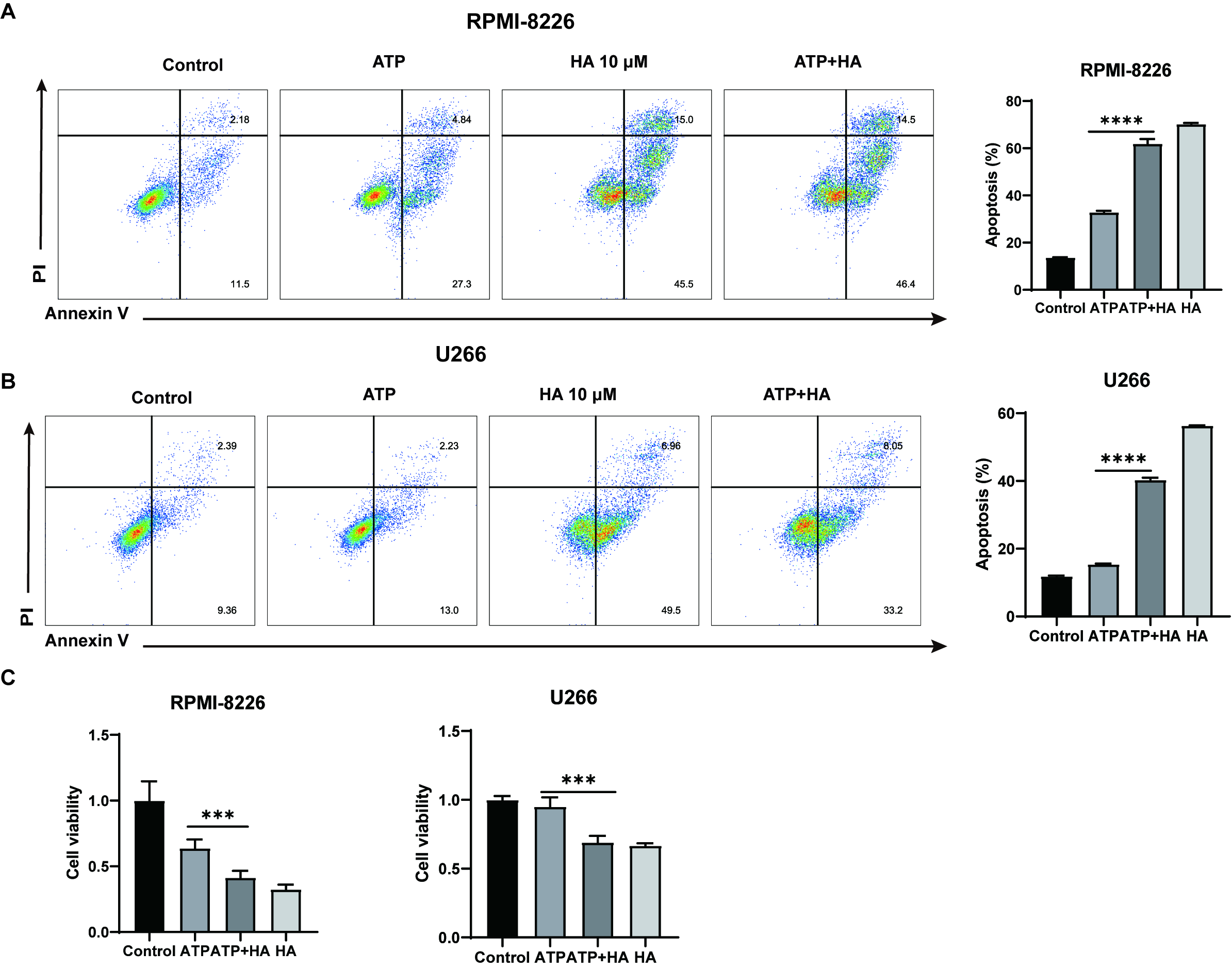


**Supplementary Fig. 6** Apoptosis assay of RPMI-8226 (A) and U266 (B) treated with HA or in the presence of ATP at 48h. The percentage of apoptotic cells (Annexin-V+PI+) after treatment was analyzed by the flow cytometry. (C)Detecting the cell viability of RPMI-8226 and U266 treated with HA in the presence of ATP at 48h. Data are presented as the mean ± SD of at least three independent experiments, and comparisons were evaluated by two-tailed Student’s t test. ***, *P* < 0.001, ****, *P* < 0.0001.

**Table S1. Specific primers used for qRT-PCR**

| Primer | Sequence (5’-3’) |
| --- | --- |
| LGMN-F  LGMN-R  CTSL1-F  CTSL1-R  ACP5-F  ACP5-R  TPP1-F  TPP1-R  CTSF-F  CTSF-R  CTSB-F  CTSB-R  GAPDH-F  GAPDH-R  NPC2-F  NPC2-R  GM2A-F  GM2A-R  CTNS-F  CTNS-R | ATCGTGGCAGGTTCAAATGG  GGACTCCCTGATAGACATCTGTG  CTTTTGCCTGGGAATTGCCTC  CATCGCCTTCCACTTGGTC  GACTGTGCAGATCCTGGGTG  GGTCAGAGAATACGTCCTCAAAG  CCTCCACACGGTGCAAAAATG  CTCTGCTTGTCGGATGCTCAG  AGCCCAAGTCAGCCTTCAC  CGCACCATGTTATTGACAAAGAC  GAGCTGGTCAACTATGTCAACA  GCTCATGTCCACGTTGTAGAAGT  GGAGCGAGATCCCTCCAAAAT  GGCTGTTGTCATACTTCTCATGG  TCCTGGCAGCTACATTCCTG  ACAGAACCGCAGTCCTTGAAC  AGCCATCCCAGCTCAGTAG  GGCTCCCCAGTAGGAATTAACA  TCCTCCTGTCGTAAAGCTGGA  GCCGGTCTGATTGGAGTGAT |
| TFEB-F | ACCTGTCCGAGACCTATGGG |
| TFEB-R | CGTCCAGACGCATAATGTTGTC |
| TFE3-F | CCGTGTTCGTGCTGTTGGA |
| TFE3-R | GCTCGTAGAAGCTGTCAGGAT |

**Table S2. The shRNA sequences**

| Homo-TFE3-sh1: | CCGGCAGCTCCGAATTCAGGAACTACTCGAGTAGTTCCTGAATTCGGAGCTGTTTTTGAATT |
| --- | --- |
| Homo-TFE3-sh1: | CCGGATTGTTGCTGACATAGAATTACTCGAGTAATTCTATGTCAGCAACAATTTTTTGAATT |
| Homo-TFE3-sh1: | CCGGGCCTGGAGTCCAGTTACAATGCTCGAGCATTGTAACTGGACTCCAGGCTTTTTGAATT |

**Table S3. The Antibodies**

| **ANTIBODIES** | **SOURCE** | **IDENTIFIER** |
| --- | --- | --- |
| LAMP1 | Proteintech | Cat# 21997-1-AP;  RRID: AB_2878966 |
| TFE3 | Proteintech | Cat# 14480-1-AP;  RRID:  AB_2199587 |
| NHE1 | Proteintech | Cat #: 67363-1-Ig  RRID:  AB_2882615 |
| mTOR | Cell Signaling Technology | Cat# 2983;  RRID: AB_2105622 |
| Phospho-mTOR (Ser2448) | Cell Signaling Technology | Cat# 5536;  RRID: AB_10691552 |
| Tubulin | Proteintech | Cat# 11224-1-AP;  RRID: AB_2210206 |
| GAPDH | Proteintech | Cat# 60004-1-Ig;  RRID: AB_2107436 |
| Anti-rabbit IgG (H+L), F(ab')2 Fragment (Alexa Fluor 488 Conjugate) | Cell Signaling Technology | Cat#4412;  RRID: AB_1904025 |

**Table S4.** Characteristics of MM patients used in this study

| Patient | Sex | Age | ISS | M-protein | Myeloma cells in BM (%) | Creatinine (μmol/L) | Serum Albumin (g/L) | LDH | β2-MG  (ng/ml) |
| --- | --- | --- | --- | --- | --- | --- | --- | --- | --- |
| #1 | F | 58 | Ⅲ | 4.9 | 19.5 | 659 | 37.4 | 166 | 12.84 |
| #2 | F | 74 | Ⅲ | 27.782 | 52 | 76 | 37 |  | 2.169 |
| #3 | M | 61 | Ⅲ | 34.1088 | 42 | 819 | 31.1 | 167 | 7.761 |
| #4 | M | 67 | Ⅲ | 45.0254 | 50.5 | 97 | 30.9 | 315 | 4.937 |
| #5 | M | 79 | / | 21 | 35.5 | 215 | 27.2 | 106.1 | / |
| #6 | M | 56 | Ⅰ | 8 | 15 | 50.4 | 35 | 137.2 | 2.49 |
| #7 | F | 60 | Ⅲ | 11 | / | 334.7 | 43.7 | 232.9 | 13.07 |
| #8 | M | 58 | Ⅲ | 0 | 89.5 | 106.2 | 44.4 | 147.8 | 6.56 |
| #9 | M | 59 | Ⅲ | 57 | 52 | 95.9 | 29.8 | 129.9 | 6.83 |
| #10 | M | 78 | Ⅱ | 51.56 | 61.5 | 111.3 | 28.8 | 118 | 5.35 |
