# Supplementary figures and images for: Hexamethylene amiloride induces lysosome-mediated cell death in multiple myeloma through transcription factor E3
Source: Cell Death Discov. 2024 Dec 18;10:505. doi: 10.1038/s41420-024-02269-9 (PMC11655536; doi:10.1038/s41420-024-02269-9)

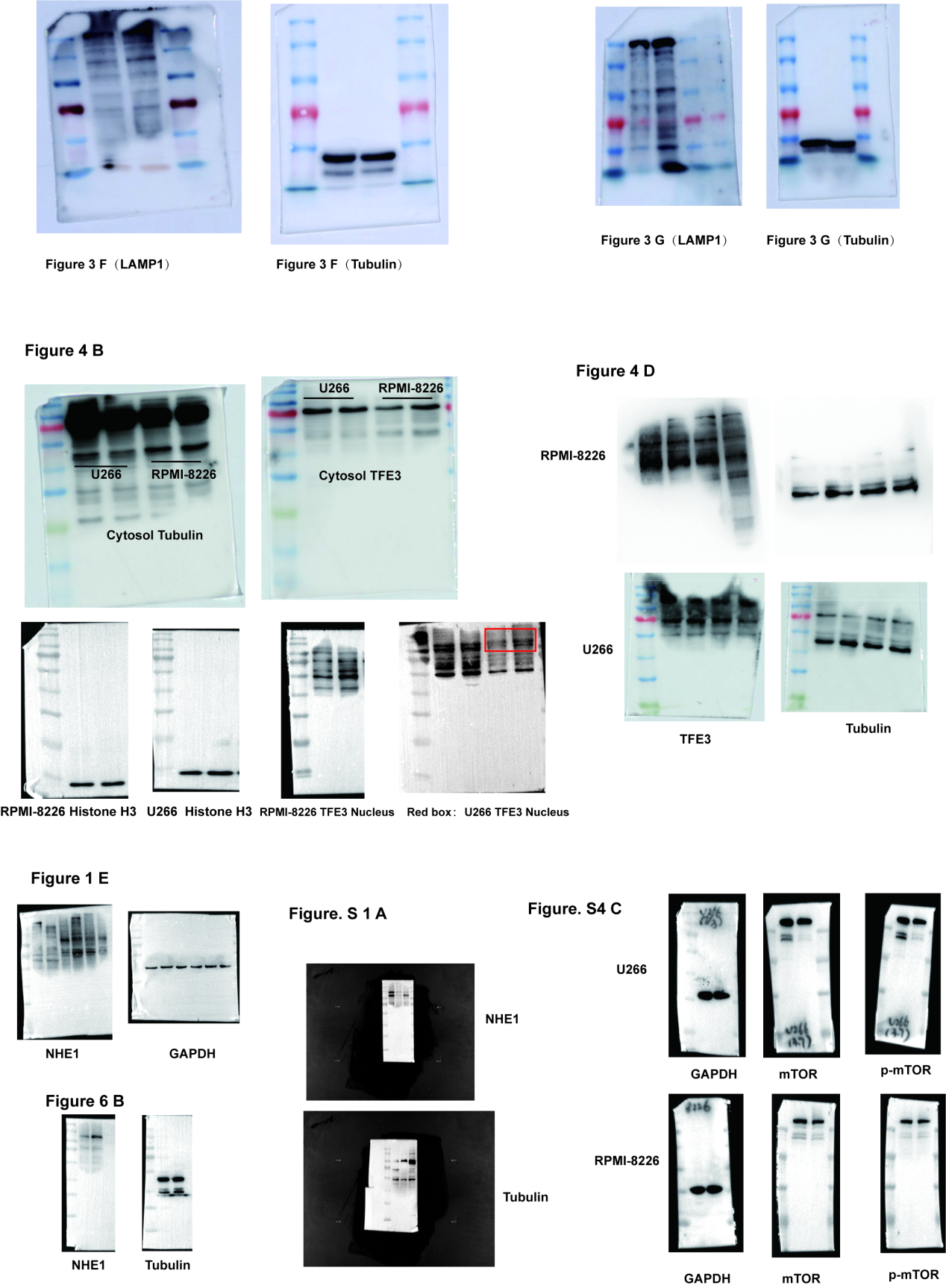

Supplement: Supplementary file 2 — Supplemental Material [file 41420_2024_2269_MOESM2_ESM.docx]
